# Empirical Bayesian significance measure of neuronal spike response

**DOI:** 10.1186/s12868-016-0255-x

**Published:** 2016-05-21

**Authors:** Shigeyuki Oba, Ken Nakae, Yuji Ikegaya, Shunsuke Aki, Junichiro Yoshimoto, Shin Ishii

**Affiliations:** Graduate School of Informatics, Kyoto University, Yoshida-Honmachi, Kyoto, Japan; Graduate School of Pharmaceutical Sciences, University of Tokyo, Tokyo, Japan; Graduate School of Information Science, Nara Institute of Science and Technology, Ikoma, Japan

**Keywords:** Functional connectivity of neurons, Spike statistics, Generalized linear model, Empirical Bayesian testing

## Abstract

**Background:**

Functional connectivity analyses of multiple neurons provide a powerful bottom-up approach to reveal functions of local neuronal circuits by using simultaneous recording of neuronal activity. A statistical methodology, generalized linear modeling (GLM) of the spike response function, is one of the most promising methodologies to reduce false link discoveries arising from pseudo-correlation based on common inputs. Although recent advancement of fluorescent imaging techniques has increased the number of simultaneously recoded neurons up to the hundreds or thousands, the amount of information per pair of neurons has not correspondingly increased, partly because of the instruments’ limitations, and partly because the number of neuron pairs increase in a quadratic manner. Consequently, the estimation of GLM suffers from large statistical uncertainty caused by the shortage in effective information.

**Results:**

In this study, we propose a new combination of GLM and empirical Bayesian testing for the estimation of spike response functions that enables both conservative false discovery control and powerful functional connectivity detection. We compared our proposed method’s performance with those of sparse estimation of GLM and classical Granger causality testing. Our method achieved high detection performance of functional connectivity with conservative estimation of false discovery rate and q values in case of information shortage due to short observation time. We also showed that empirical Bayesian testing on arbitrary statistics in place of likelihood-ratio statistics reduce the computational cost without decreasing the detection performance. When our proposed method was applied to a functional multi-neuron calcium imaging dataset from the rat hippocampal region, we found significant functional connections that are possibly mediated by AMPA and NMDA receptors.

**Conclusions:**

The proposed empirical Bayesian testing framework with GLM is promising especially when the amount of information per a neuron pair is small because of growing size of observed network.

**Electronic supplementary material:**

The online version of this article (doi:10.1186/s12868-016-0255-x) contains supplementary material, which is available to authorized users.

## Background

Connectomics, which seeks to identify the connectivity structure between all pairs of neurons not only in local circuits but over the entire brain, is a crucial research direction [1], because the brain’s functions are believed to emerge within the connectivity structure of its constituent elements, the neurons. Toward such a direction, functional connectivity analyses provide a powerful bottom-up tool to investigate the neurophysiology of the relationships of multiple neurons [2–4]. Moreover, a decoding study has quantified information amount that is encoded into functional connectivity of retinal ganglion cells [5]. When we focus on information processing principles of local neuronal circuits, we need a sophisticated approach to unify top-down simulations and bottom-up experimental observations both in terms of functional connectivity and anatomical connectivity. The reliable detection of functional connectivity is thus vital to identify the functions of local and global networks in the brain. Similar attempts have focused on the functional connectivity of the higher-order view of the brain: dynamic causal modeling of the brain regions [6], for example. Such research is sometimes called macro-connectomics. Although our functional connectivity analysis method can be applied to such macro-connectome problems, in this study we focus on functional connectivity between neurons, one of the elementary levels of the brain’s hierarchy.

By definition, a pair of neurons is called functionally connected if the physiological activities of these neurons interact with each other. Granger causality provides a theoretical definition to a directed causal relationship that can be estimated from time course observations like spike trains from neuronal circuits [7]. In contrast, structural connectivity implies anatomical observation of synaptic structures between the neurons found by electron microscopy or the imaging of synaptic markers [8]. Functional connectivity and structural connectivity have different theoretical implications. Structural connectivity does not necessarily imply functional connectivity; since most synapses are silent or inactive in vivo, few active synapses establish functional connectivity [9, 10]. Functional connectivity does not necessarily require structural connectivity; since the observation cannot be complete, functional connectivity may be produced by indirect causality bypassed by unobservable neurons and glial cells and mediated by various transmission channels like electronic coupling and metabolic factors. Some studies have shown indirect evidence of relationships between functional and structural connections [8]. Because one major objective of the physiology of neuronal circuits is to determine their functions, functional connectivity analysis can be more important than its structural counterpart.

In order to estimate functional connectivity among many neurons, we require sufficient data of electrical activity of the neurons. Recent functional multi-neuron calcium imaging technique enabled us to observe hundreds or thousands of neurons’ activities simultaneously [11]. However, there are following two trade-offs between information amounts that can affect the quality of functional connectivity estimation. The first is a trade-off due to limited observation speed of a scanning microscopy; we can observe the larger number of neurons by the wider field of sight with the larger spatial resolution, leading to the lower temporal resolution. The second is a trade off due to unavoidable photobleaching; higher signal to noise ratio requires stronger light emission, leading to stronger photobleaching of fluorescent dyes that shorten the observation time length. Thus, these two trade-offs limit information amount per neuron even when the number of simultaneously recorded neurons is large. Moreover, information amount per a pair of neurons is decreasing with growing number of neurons because the number of pairs of neurons is quadratic to the number of neurons. We call this situation an information shortage for functional connectivity estimation.

In the current study, we focus on generalized linear models (GLMs) of spike response functions (SRFs) and their extensions, which are found powerful statistical tools to sketch the functional connectivity between multiple neurons [4, 7, 12–16]. An SRF expresses the increase and decrease of the spike generation probability triggered by a pre-synaptic spike input as a function of the lag-time between pre-synaptic input and post-synaptic output spikes. Based on the GLM framework, SRFs for all possible pairs of neurons are estimated simultaneously, and the Granger causality test substantially reduces false positives caused by indirect causality that cannot be distinguished if they are estimated for each local pair of neurons. Recently, several studies have presented sparse estimation methods that prefer zero connection weights for a large part of all neuron pairs to improve the estimation of GLM-based SRFs [12, 13, 15, 16]. On the other hand, there have been a few attempts of network structure inference without focusing on fitting GLM, such as information theoretic analysis of Granger causality [17] and inference of Dynamic Bayesian Networks [18].

In case of information shortage, two major requirements to estimate SRFs, model fitting and detection accuracy, are not compatible with each other. Estimation of GLM, in case of information shortage, owes its performance much to regularization term, in which regularization coefficient is tuned for better model fitting. Recent sparse estimations of GLM are not exceptions. However, the best-tuned coefficient for model fitting does not generate the best results for detection accuracy; not only that there is no guarantee of compatibility, but also that there is a severe contradiction. Discrimination accuracy by the sparse estimation of SRFs usually depends on the arbitrary tuning of a hyperparameter that controls the sparseness. The hyperparameter tuning, however, involves severe problems that are not well understood. Namely, there is no guarantee that the best hyperparameter to minimize the fitting error in terms of cross-validation leads to the best discrimination accuracy to detect functional connectivity. Actually, the connectivity detection performance is not favorable when we tune the sparse model so as to maximize the likelihood. Moreover, no good method exists to control false positives with sparse estimation mainly because it is difficult to obtain closed-form null distributions of testing statistics.

There have been many studies focusing on model fitting, but few studies focusing on detection accuracy except for the likelihood-ratio testing of Granger causality [7]. However, they did not consider the case of information shortage and did not include any regularization factors. The performance of their estimation might be sub-optimal, particularly with information shortage.

In this study, we propose a new functional connectivity analysis method focusing on improving the detection performance especially in the case of information shortage. Our method consists of two major procedures. In the first, SRFs are identified by estimating the parameters of GLMs with smooth bases to represent SRFs and L2 regularization. The second procedure is a Granger causality analysis based on empirical Bayesian testing. Because our situation for functional connectivity analysis is a typical multiple simultaneous testing, false positive control is definitely important. Empirical Bayesian testing provides a reliable way to stably control the false positive proportion.

Our research scope is summarized in Table 1. We focus on the case with information shortage in which the number of neurons is large but the observation length is relatively short. Because the amount of information available to estimate SRF is proportional to the number of spikes per neuron pair, the statistical uncertainty is large in various imaging studies, like calcium imaging. Thus, we propose a methodology that is essentially different from best practice in electrophysiological studies. Namely, we apply multiple testing to determine the functional connectivity, rather than to estimate SRF, for each pair of neurons. The goal is to list a set of candidate connections while controlling false positives, rather than to build a single most likely model that maximizes the fitness or expected likelihood. Toward this goal, we apply a GLM model with non-sparse regularization in contrast to one with sparse regularization.

**Table 1:** Conceptual difference between two distinct typical cases of functional neuronal connectivity analyses

	Long	Short
Typical case	Multiple electrodes	Calcium imaging
No. of neurons	Small	Large
No. of spikes per a pair of neurons	Large	Small
Methodology	Sparse estimation	Multiple testing
Goal	Single most likely model	Set of candidate connections
Criterion	Fitness (likelihood)	False discovery rate
Model	GLM (sparse)	GLM (non-sparse)

## Spike response estimation

### Generalized linear model of spike responses

In this section, we introduce a formulation of GLM of spike responses according to [7, 12, 16].

Suppose that we have a spike train dataset, ${\mathbf {N}}(1 : T)=\{N_i(t): i=1,\ldots ,C; t=1,\ldots ,T\}$, where $N_{i}(t)$ denotes the number of spikes of the *i*th neuron ($i=1,\ldots ,C$) in the *t*th time-bin ($t=1,\ldots ,T$) with a common width over all the bins. We assume the bin width is short enough so that the maximum spike number in a single bin is unity, and thus $N_{i}(t)$ takes either one or zero. We also assume that *C* neurons may receive a common input signal transmitted through *L* external channels: ${\mathbf {E}}(1:T)=\{E_l(t)\in {{\mathbb {R}}}: l=1,\ldots ,L\}$, where $E_l(t)$ denotes a real-valued signal sent through the *l*th channel.

A stochastic spike response model represents the conditional probability to observe a spike of the *i*th neuron in the *t*th time bin $p_{i}(t) \equiv {\mathrm{Pr}}( N_i(t)=1 | {\mathbf {N}}(1:t-1), {\mathbf {E}}(t), {\mathbf {R}}_i )$, where conditions ${\mathbf {N}}(1:t-1)$, ${\mathbf {E}}(t)$, and ${\mathbf {R}}_i$ denote the past spike history up to time $t-1$ of all neurons $c=1,\ldots ,C$ including the *i*th neuron itself, the external input at time *t*, and a set of parameters that define the response function of the *i*th neuron, respectively. According to GLM, the conditional probability is a function of total spike response $\lambda _{i}(t)$,

1 $$\begin{aligned} p_{i}(t) = f( \lambda _{i}(t) ), \end{aligned}$$

where the total spike response is given as a linear summation of all external inputs $E_l(t) (l=1,\ldots ,L)$ and the past spikes from all neurons ${\mathbf {N}}(t-M:t-1)$,

2 $$\begin{aligned} \lambda _{i}(t)& \equiv \lambda _{i}(t; {\mathbf {N}}(t-M:t-1), {\mathbf {E}}(t), {\mathbf {R}}_i ) \\ &\quad =R_{i0} + \sum _{l=1}^L R^{{\mathrm{E}}}_{il} E_l(t) \\&\quad + \sum _{c=1}^C \sum _{s=1}^M R_{ic}(s)N_c(t-s). \end{aligned}$$

$R_{i0}$, $R^{{\mathrm{E}}}_{il}$, and $R_{ic}(s)$ are respectively a background activity level of the *i*th neuron, a response coefficient to the *l*th external input, and the spike response with lag-time *s* to the *c*th neuron, of the *i*th neuron. Natural number *M*, called a window, is the maximum time-lag considered in the history. *f*(*x*) is a (possibly non-linear) link function from total response $\lambda _i(t)$ to probability $p_i(t)$, and we concentrate on a case of logistic link $f(x)=1/(1+\exp (-x))$. A reasonable alternative may be $f(x)=1-\exp (-\exp (x))$, called the *complementary log–log link function*. The latter case makes this spike response model equivalent to a Poisson spike model at a limit of infinitesimal bin-width [12].

We may consider common external input ${\mathbf {E}}(t)$ that effectively represents the total effect received by the observable neurons from unobserved ones and/or other external inputs. If we can directly observe those external inputs, the estimation of the spike response model is reduced to an auto-regression (AR) problem; by regarding glial activities as observable external inputs to a neuronal network, a GLM-based spike response model for a neuron-glia system can be estimated [19]. If we cannot observe the external inputs, the estimation is of an AR type with a moving average (ARMA), in which we need to simultaneously estimate the external inputs and the response functions of individual neurons.

### Estimation and errors

We may obtain a small but non-zero estimation of response functions for some pairs of neurons that in fact have no functional connectivity because of the finite amount of available data. Such limited data causes statistical uncertainty preventing a clear-cut determination and two types of statistical errors, false negatives and false positives.

To deal with such statistical uncertainty in our application of functional connectivity analysis, we have several options in each of the following two steps that constitute functional connectivity analysis: (a) the estimation method of a GLM-based spike response model including spike response $R_{ic}(s)$ between neurons *i* and *c*, and (b) the selection of statistics to test the null hypothesis, where the spike response $R_{ic}(s)$ is zero for any time-lag *s* if the null hypothesis is true, and $R_{ic}(s)$ is not zero for some *s* if the alternative hypothesis is true. Our ways of dealing with these two issues will be discussed in the following subsections.

### Application of smooth bases and regularization term

This subsection describes a way to estimate a GLM-based spike response model.

We assume that each spike response function, $R_{ic}(s)$, is represented as a linear combination of a small number of smooth bases:

3 $$\begin{aligned} R_{ic}(s) = \sum _{k=1}^K A_{ick} B_k( s ), \end{aligned}$$

where $B_k(s)$ and $A_{ick}$ denote the *k*th basis function that is shared by all neurons and the *k*th basis loading coefficient between neurons *c* and *i*, respectively. To reflect the consistently positive (negative) character of the facilitatory (suppressive) post-synaptic current, EPSC (IPSC), and the consistent profile of EPSC (IPSC) induced by a single pre-synaptic spike, each basis function is given by the following Gamma density function:

4 $$\begin{aligned} B_k( s ) = {\mathrm{Ga}}( s ;m_k, v_k ). \end{aligned}$$

Here, ${\mathrm{Ga}}( s; m_k, v_k )$ denotes the probability density function of a Gamma distribution whose mean and variance are set at $m_k=v_k=\frac{1}{2}k^2$. The Gamma density function as a filter basis was first proposed in [20]. A basis function with a small index *k* peaks at a small *s* value, which induces a large but short-delayed EPSC or IPSC, and that with a large index is broad with a large mean value, which induces a small but long-lasting EPSC or IPSC. Number of bases *K* is arbitrarily determined so that the GLM model fits the real spike response well. Smoother response function is preferred in the estimation when a smaller number *K* is set, which can improve the estimation by preventing over-fitting to statistical uncertainty especially in cases with information shortage.

From Eqs. (2) and (3), the total spike response of the *i*th neuron is given by

5 $$\begin{aligned} \lambda _{i}(t) &=R_{i0}+\sum _{l=1}^L R_{Eil} E_l(t) \\&\quad +\sum _{c=1}^{C} \sum _{k=1}^K \sum _{s=1}^M A_{ick} B_k(s) N_c(t-s). \end{aligned}$$

When estimating the spike response model parameters, the following regularized log-likelihood function is independently maximized with respect to the unknown parameters $R_{i0}$, $R_{Eil}$, and $A_{ick}$, $l=1,\ldots ,L, c=1,\ldots ,C, k=1,\ldots ,K$, for each neuron *i*:

6 $$\begin{aligned} L^{i} &=\sum _{t=1}^T L^i_t - \eta {\mathrm{Reg}}^i, \end{aligned}$$

where

$$\begin{aligned} L^i_t & \equiv N_i(t) \log ( f( \lambda _{it}) ) \\&\quad + (1-{N_i(t)})\log (1-f( \lambda _i(t) )) \end{aligned}$$

is log-likelihood at time *t*, $\eta$ is a hyperparameter that controls the strength of regularization, and ${\mathrm{Reg}}^i$ is the regularization term. We may use the following L2 regularization to encourage non-sparse estimation of the parameter $A_{ick}$:

7 $$\begin{aligned} {\mathrm{L2:}} \quad {\mathrm{Reg}}^i \equiv \frac{1}{2}\sum _{c=1}^{C}\sum _{k=1}^K A_{ick}^2. \end{aligned}$$

We alternatively consider the following L1 or group lasso regularization for facilitating sparse estimation:

8 $$\begin{aligned} {\mathrm{L1:}}\quad {\mathrm{Reg}}^i \equiv \sum _{c=1}^{C} \sum _{k=1}^K \left| A_{ick}\right| , \end{aligned}$$

9 $$\begin{aligned} {\mathrm{GL:}}\quad {\mathrm{Reg}}^i \equiv \sum _{c=1}^{C} \sqrt{ \sum _{k=1}^K A_{ick}^2}. \end{aligned}$$

The common input term $E_l(t)$ in our GLM-based spike response model (2) is fixed at an estimated value in a preprocess (“Estimation of external input” section) prior to the model estimation. The parameter estimation to maximize the regularized likelihood function (6) is implemented using a dual augmented Lagrangian method [21].

### Estimation of external input

When some common external inputs $E_l(t), l = 1,\ldots ,L$ were supposed to exist but not given, they were estimated using principal component analysis (PCA) [22] of the observed time course. Let us imagine a typical case that there are some synchronized spikes of many observed neurons. In this case, we would detect facilitative connectivity between all neurons that emit the synchronized spikes. However, we can reduce the effect of the synchronized spikes to the estimation of functional connectivity between the observed neurons if we regard the synchronized spikes as an effect of common external input. We can extract the synchronized signal as the first principal component of a set of smoothed spike sequences of the neurons, or multiple signals as some components if there are some distinct sets of neurons that are synchronized. Note that we assume these common external inputs as a minimum device to reduce the harmful effect to functional connectivity estimation, rather than to infer any external reality.

We first applied a moving average filter of a certain window size to the spike time course $N_i(t)$ of all the observed neurons $i=1,\ldots ,C$ to obtain smoothed spike density profiles. Next, we calculated *L* principal components with eigenvalue decomposition of $C \times C$ covariance matrix for the smoothed spike density profiles of the *C* observed neurons and regarded them as the estimated *L* external inputs. Finally, we fit the GLM model involving the common external inputs and assessed the fitness using Akaike’s information criteria (AIC) [22] to determine the number of principal components *L*, where AIC is defined as the log-likelihood value minus the number of free parameters of the GLM.

If there is no external input, the smoothed spike density is based on the internal fluctuation stemming from the constant term [$R_{i0}$ in Eq. (2)] of the observed neurons. Then there is no significant principal component of the smoothed spike density profiles. If there are principal components of the smoothed spike density profiles, they cannot be explained by the bias of the spontaneous activities of the observed neurons; therefore, they are based on the external inputs. Estimation of the external inputs by the above method corresponds to estimation of slow behaviors of the network and is useful for removing factors that are not able to be represented by our GLM; it is beneficial to our main objectives of estimating the spike response functions and their significance measure in a stable manner.

## Statistical significance

Here, we focus on statistical tests for functional connectivity analyses of GLM-based spike response models. Our statistical tests are based on Granger causality, but there are several options when applied to GLM-based spike response models.

### Granger causality and a corresponding test statistic

Granger causality is a criterion to determine the existence of a directed causal relationship between two nodes, from both of which we observed time sequences. By definition, it is said that there is Granger causality from nodes A to B if we can significantly better predict the future time sequence of node B using the information of the past and current time sequences of nodes A and B than only using that of node B. In the context of the functional connectivity analysis of our GLM-based spike response models, the Granger causality from the *c*th to the *i*th neurons can be examined by hypothesis testing with null hypothesis $H_0^{(i,c)}: R_{ic}(s)=0$ for all $s=1,\ldots ,M$ against alternative hypothesis $H_1^{(i,c)}: R_{ic}(s)\ne 0$ for some $s=1,\ldots ,M$.

Kim et al. [7] proposed a likelihood-ratio test of all $H_0^{(i,c)}, i,c=1,\ldots ,C$ by a log-likelihood-ratio statistic:

10 $$\begin{aligned} {\mathrm{LR}}^{ic} = -2( \ln L_0^{ic} - \ln L^{i} ), \end{aligned}$$

where $L_0^{ic}$ and $L^{i}$ are the log-likelihood of the null and alternative hypotheses, respectively. This statistic reflects the loss of model fitness by omitting the functional connectivity from neuron *c* to neuron *i*; the alternative likelihood $L^{i}$ is first calculated for the post-synaptic neuron *i* and the null likelihood $L_0^{ic}$ is calculated by omitting the contribution of each of the pre-synaptic neurons $c=1,\ldots ,C$. In their likelihood-ratio test, the alternative log-likelihood is given by Eq. (6) without the L2 regularization, that is, with $\eta = 0$, and the null log-likelihood is given by Eq. (6) with $R_{ic}(s)=0$ for all $s=1,\ldots ,M$ (and $\eta = 0$). Note that the null log-likelihood depends on the pre-synaptic neuron index *c*, but the alternative log-likelihood does not, because the null likelihood has an additional constraint dependent on the pre-synaptic neuron index, which is not involved in the alternative likelihood.

We can calculate the p value of the likelihood-ratio test based on the fact that the test statistic ${\mathrm{LR}}^{ic}$ obeys a chi-squared distribution with *M* degree of freedom in a large sample limit [23]. Considering the situation of multiple hypothesis testing, q values were also calculated based on the p values for all $H_0^{(i,c)}, i,c=1,\ldots ,C$ based on the standard procedure [24].

Kim et al.’s study [7] followed the standard methodology of statistics; especially for a relatively small amount of data, however, parameter estimation without any regularization may suffer from failure or instability, producing an unreliable calculation of the likelihood-ratio. One possible way to overcome this difficulty is to introduce regularization to the null and alternative likelihood functions, but then the test statistic no longer obeys the asymptotic chi-square distribution. Moreover, parameter estimation for the null likelihood must be performed for every pair of pre- and post-synaptic neurons, which is computationally expensive.

### Empirical Bayesian testing

Empirical Bayesian testing uses empirical null samples in place of null distributions that are analytically defined like the one described in the previous sub-section. In our particular application, we do not know the null distribution analytically because of the introduction of regularization and kernel functions to the GLM-based spike response models; in this case, empirical Bayesian testing is a practical choice.

In our empirical Bayesian testing, a certain number of empirical null samples of test statistics are obtained by the following surrogation method. We assume several surrogate neurons, each of which emits an artificial spike train, by time-shifting a real neuron’s spike train: $N_{c^*}(t)=N_c(t+T_S)$ for some $c\in {1,\ldots ,C}$. Here, we also assume that there is no prominent periodic activity in the network and set the time-shift $T_S$ to a number larger than the time-lag *M*. Then, the spike train of a surrogate neuron $c^*$ becomes independent from that of any real neuron $i=1,\ldots ,C$, in the time lag. After adding a certain number of surrogate neurons to the set of real neurons, we estimate the GLM-based spike response model. In this estimated spike response model, the test statistic between a real neuron A and a surrogate neuron C, which was generated by time-shifting the spike train of a real neuron other than A, can be regarded as a null test statistic, because A and C are independent, with no functional connectivity between them.

Note that there is a non-zero probability to include some statistic values from the non-independent (false negative) pairs of neurons in the surrogating process, which can weaken detection power. However, the risk is small because the strength of ordinary response is negligible for a time lag larger than a certain limit and the risk can be smaller by setting large enough time-shift for the surrogate. More importantly, inclusion of false negatives does never violate conservativeness.

In multiple simultaneous hypothesis testing like functional connectivity analysis, q value, which is an estimation of false discovery rate, is often used as an alternative significance measure to a p value [24].

When test statistics are available along with empirical null samples for calculating them, q values can be estimated directly from them without calculating the p values. In our empirical Bayesian multiple simultaneous hypothesis testing, we used this direct method to obtain q values for all the multiple hypotheses. This procedure is described in the Additional file 1.

### Shape-related statistics

According to empirical Bayesian testing, we can use an arbitrary test statistic. We also examined the following statistics since they are easier to compute compared to likelihood-ratio statistic after estimating the GLM-based spike response model, but they still represent the non-zero character of the spike response function.

We prepared several statistics to characterize the spike response function (3) between neurons *c* and *i*.

*Surface*

$$\begin{aligned} S_{ic}^{{\mathrm{Surface}}} \equiv \sum _{s=1}^M |R_{ic}(s)| \end{aligned}$$

*Peak*

$$\begin{aligned} S_{ic}^{{\mathrm{Peak}}} \equiv {\mathrm{Max}}_s |R_{ic}(s)| \end{aligned}$$

*Parameter vector norm with a special metric design (MD)* We evaluate the norm of parameter vector $A_{ic\cdot }$ with a special metric design $\Sigma _{kk'}$.

$$\begin{aligned} S_{ic}^{{\mathrm{MD}}} \equiv \sqrt{ \sum _{k,k'} A_{ick} A_{ick'} \Sigma _{kk'} }, \end{aligned}$$

where $\Sigma _{kk'}$ is the ($k,k'$)-th element of a regularized inverse of the following covariance matrix ${\mathbf {S}}_0$ of the parameter vectors of null links

$$\begin{aligned} {\mathbf {S}}_0 \propto \sum _{(i,c)\in {\mathrm{H_0}}} A_{ic\cdot }{}^{{\mathrm {T}}}A_{ic\cdot }. \end{aligned}$$

Here, $A_{ic\cdot }$ is a *K*-dimensional parameter vector whose *k*-th element is $A_{ick}$ and ${}^{{\mathrm {T}}}$ denotes a transpose. In the equation above, the summation was taken over every pair of pre- and post-synaptic neurons whose functional connectivity was determined not to exist by the statistical test. Thus, the MD incorporates the anisotropic nature of the null distribution and evaluates the distance between each case and the typical null cases.

*Delay*

$$\begin{aligned} S_{ic}^{{\mathrm{Delay}}} \equiv {\mathrm{Argmax}}_s |R_{ic}(s)| \end{aligned}$$

The last term, **Delay**, is a bit different, because it is used for describing a functional connection detected by a certain statistical test, rather than for defining a test statistic to detect the functional connectivity.

### Statistical tests for functional connectivity analysis

In this study, we compared the following statistical tests in a scenario of functional connectivity analysis based on a GLM-based spike response model. They examine a pairwise functional connectivity, that is, whether a direct relationship exists from a pre-synaptic neuron *c* to a post-synaptic neuron *i*.

*CHI2* If there is no regularization term, a chi-square test can be applied to likelihood-ratio statistic ${\mathrm{LR}}^{ic}$, because it asymptotically obeys a chi-square distribution with *M* degree of freedom. Then, we can approximately use a chi-square test by setting the regularization hyperparameter $\eta$ to a small positive value to avoid a divergence in the optimization procedure. A p value is simply obtained by integrating the chi-square distribution. A q value is estimated using the p values of all null hypotheses $H_0^{(i,c)}$, based on a standard procedure.

*DOF* A p value and hence a q value were obtained by the same procedure as in CHI2 above, except that the degrees of freedom were determined to fit an empirical null distribution of the likelihood-ratio statistic ${\mathrm{LR}}^{ic}$. Here, the empirical null distribution was produced by the surrogation method described in “Shape-related statistics” section.

*EB* Empirical Bayesian testing was applied to the likelihood-ratio statistic after the model parameters were estimated by the optimization with a larger regularization hyperparameter $\eta$. By comparing the results of EB and CHI2 (or DOF), we can see the contribution of the L2 regularization and employing smooth bases.

*EB-arbitrary stat* Because empirical Bayesian testing can be applied to an arbitrary test statistic, the statistics described in “Shape-related statistics” section were also examined in its framework. Note that we did not apply empirical Bayesian testing for estimation results with L1 and group lasso regularization, because the empirical null distributions of the statistics in “Shape-related statistics” section were found to be quite different among neurons, which was not the case with the L2 regularization.

*Confidence interval (CI) and MaxZ* A 95 % Wald confidence interval is calculated for each estimated value of GLM weight $w_k$,

$$\begin{aligned} w_k \pm 1.96 \sigma _{kk}, \end{aligned}$$

where $\sigma _{kk}$ is an asymptotic standard deviation that is derived using Hessian of the objective function [15]. According to [15], directed connectivity is detected if any CI corresponding to the connectivity is significantly different from zero. We also consider the Z-score of a weight value $Z_k = |w_k|/ \sigma _{kk}$ and that of the response function $Z(s) = \sum _{k=1}^K Z_k B_k(s)$. We integrate them into a *MaxZ* statistic, a maximum of $Z(s), s=1,\ldots ,M$ corresponding to the directed connection, so that a criteria, ${ MaxZ} > 1.96$, is equivalent to the application of CI in [15]. As the straight application of the 95 % CI is no longer a valid multiple testing, we utilized our *EB-arbitrary stat* framework in order to include the MaxZ statistic in the following comparison study.

## Results

### Experiment 1: Poisson model simulation 

**Fig. 1:**
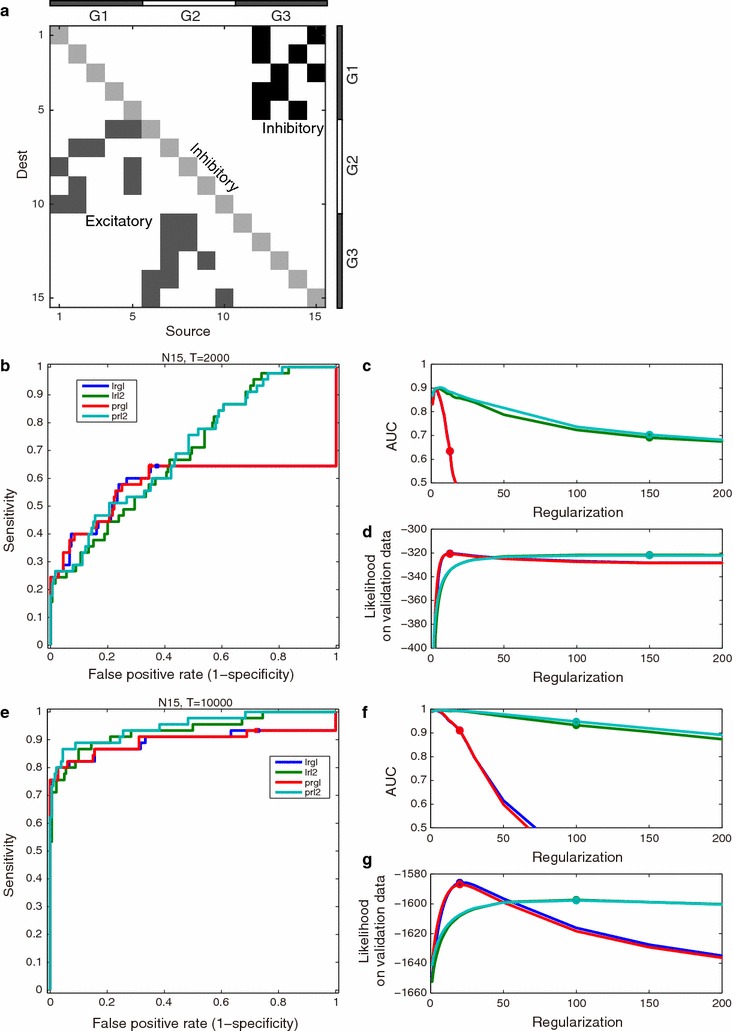
**a** Simulated neural network that was used in Experiment 1. Three groups of neurons G1, G2, and G3 construct a recurrent network: G1 $\rightarrow$ G2 $\rightarrow$ G3 $\rightarrow$ G1. *Gray scale blocks* in the matrix denote a direct functional connectivity between source (pre-synaptic) and destination (post-synaptic) neurons. **b**–**g** Performance comparison between GLMs based on sparse estimation with group lasso (gl) and on L2 regularization (l2). Comparison between logistic regression (lr) and Poisson regression (pr) is also performed. The GLM parameters were estimated with various settings of hyperparameters and the best hyperparameter value for each case was determined so as to maximize likelihood on a validation dataset. The results were evaluated with sensitivity and false positive proportion calculated by using a true directed link set for the simulation. Experiments with short ($T=2000$) and long ($T=10{,}000$) datasets are shown in the *upper* (**b**–**d**) and *lower* (**e**–**g**) sets of panels, respectively. **b**, **e** ROC curves are drawn for each case at the best hyperparameter that maximizes likelihood on the validation data. **c**, **f** AUC score for each setting of regularization hyperparameter. *Markers* denote the values at the best hyperparameter that maximizes likelihood on the validation data. **d**, **g** Accuracy of model fitting, measured by likelihood on the validation data, for each setting of regularization hyperparameter. *Markers* denote the values at the best hyperparameter that optimizes model fitting. We found that the differences were small between logistic and Poisson regression models. ROC curves or AUC were not necessarily the largest at the best-tuned values of hyperparameters for each of the four cases. Sparse estimation with the group lasso (gl) was more sensitive to the hyperparameter settings than the L2 regularization (l2)

Here, we examined the performance of the functional connectivity analysis methods using a dataset generated by simulating a network of GLM-based spike response models. We designed an artificial neuronal network of 15 neurons (Fig 1a) consisting of three groups of neurons, G1, G2, and G3, each of which consisted of five neurons. This is a recurrent network: G1 $\rightarrow$ G2 $\rightarrow$ G3 $\rightarrow$ G1. From G1 to G2, there are ten excitatory connections among 25 pairs of pre- and post-synaptic neurons. Similarly from G2 to G3, there are ten excitatory connections among 25 pairs, and from G3 to G1, there are ten inhibitory connections among 25 pairs. There is no connection between neurons belonging to the same group. The activity of each neuron was simply determined by a GLM-based spike response model, Eqs. (1) and (2), with the complementary log-log link function that simulates a Poisson spike model. Each neuron also received an external white Poisson input of around 50–150 Hz independently from the external input to the other neurons. This assumption that the external input was independent between neurons is slightly different from the one assumed by our GLM model, in which the external input is shared by the observed neurons. However, as this situation of independent Poisson inputs is much simpler, our GLM-based spike response model can deal with it by just removing the estimation procedure of the external input (“Estimation of external input” section). The activities of all 15 neurons were observed at a sampling frequency of 200 Hz. With these settings, we obtained from 0.6 to 2 spikes on average per neuron per 1 s (200 observation time-samples).

First, we compared results of the sparse and non-sparse estimation methods for GLM. We selected spike train data of 2000 and 10,000 samples from the simulated data for training and 10,000 samples for validation. The sample numbers in both cases are considerably shorter than some typical cases; as a reference, Kim et al. [7] used 50,000 samples to demonstrate their method. When using 50,000 samples, we found our method achieved 100 % accuracy, although the results are not shown here.

We compared two types of regularization, group lasso (gl) and L2 (l2) for GLM, corresponding to sparse and non-sparse estimation, respectively. We also used L1 regularization, but did not show its results because the performance with L1 was comparable to that of group lasso. We also compared two link functions, logistic (lr) and Poisson (pr); here *Poisson* stands for the complementary log-log link function that simulates Poisson spikes. The SRFs were estimated using the training data with various hyperparameters that determine the strength of regularization. The estimated SRFs were evaluated with two criteria, detection accuracy of the true functional connectivity and fitness to unseen data; detection accuracy is shown as a receiver operating characteristic (ROC) curve and the area under the curve (AUC) score, whereas fitness is given by the log-likelihood for the 10,000 samples for validation. In sparse estimation like with group lasso regularization, some statistics like *Peak* may be exactly zero for some connections. Although a naive threshold to detect functional connectivity based on such statistics would be zero, setting a non-zero threshold can in fact produce better functional connectivity estimations. Therefore, we evaluated ROC and AUC by changing the threshold value even for sparse estimation methods.

Figure 1 shows the results. In all aspects, logistic and Poisson link functions did not show any significant differences whereas the difference between sparse and non-sparse estimation was fairly large. When the hyperparameter was determined to increase the fitness to the validation data, the connectivity detection performance was poor as shown in the ROC curves (b) and (e). As shown in the panels (c) and (f), the best hyperparameters in terms of the detection accuracy or AUC were smaller than those for the largest fitness (marked). The AUC could be made larger by arbitrarily setting the hyperparameter to a smaller value. With respect to the fitness, the best model was achieved by group lasso in both sizes of training data, 2000 and 10,000 as shown in (d) and (g), respectively. With respect to the detection accuracy, the best AUC with the L2 regularization (AUC=0.88) was better than that with group lasso (AUC=0.85) for $T=2000$, and the best AUCs were 1.0 with both L2 and group lasso for $T=10{,}000$ as shown in (c) and (f), respectively. More importantly, L2 regularization performed well for a wide range of hyperparameters, while group lasso with non-optimal hyperparameters performed noticeably worse than the best performance.

We then compared the results obtained by the three statistical tests, **CHI2**, **DOF**, and **EB**, described in “Statistical tests for functional connectivity analysis” section. The GLM-based spike response model was estimated to maximize the L2 regularized log-likelihood (6). When performing the **CHI2** and **DOF**, the regularization hyperparameter was set at $\eta =0.001$. When performing the **EB**, we compared two cases **EB-LR** and **EB-regularized-LR**, in which the regularization hyperparameter was set to $\eta =0.001$ and $\eta =3.0$, respectively. The value $\eta =0.001$ was arbitrarily determined to obtain similar results to those of $\eta =0$ but with computational stability. The value $\eta =3.0$ was also arbitrarily set to a small positive value, because there was no predominant way to set it for enlarging the detection power. We also used smooth bases in the **EB-regularized-LR**.

**Fig. 2:**
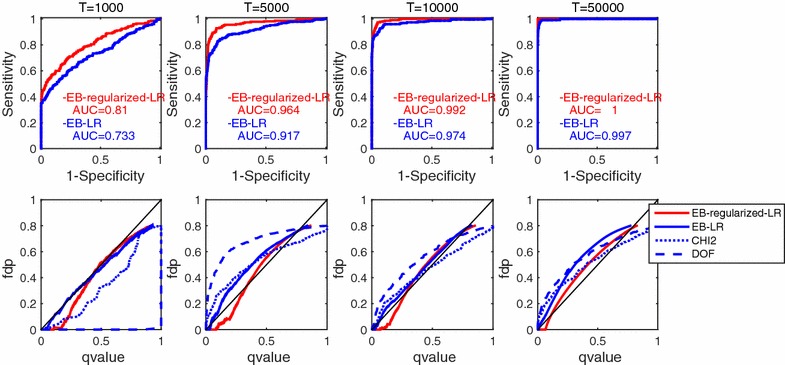
Results of Experiment 1 in which total error and FDP control were compared using several statistical testing methods. *Upper panels* show receiver operating characteristic (ROC) curves, where *horizontal* and *vertical axes* denote the specificity and sensitivity of the statistical test, respectively. *Red* and *blue* *lines* show the **EB-regularized-LR** (with regularization) and **EB-LR** (without regularization) results, respectively. In the title of each column, observation length *T* and the area under the ROC curve (AUC) scores of the two methods are shown. *Lower panels* display false positive control, where *horizontal* and *vertical axes* denote q value threshold and false discovery proportion (FDP) when the q value threshold on the horizontal axis was used, respectively. Because the q value threshold is regarded as an estimation of the FDP value, when FDP is located under and over the* thin black diagonal line* of $x=y$, the corresponding q value estimations are considered conservative and aggressive, respectively. A conservative line is preferable, because by definition, the q value should be a conservative estimation of the FDP value. If the line is close to the diagonal line, the q value estimation is faithful. *Blue broken*, *dotted*, and *solid lines* represent the q values estimated by **CHI2-LR**, **DOF-LR**, and **EB-LR** for the likelihood-ratio statistics without regularization (in fact, a small regularization was applied to avoid optimization divergence, see “Shape-related statistics” section, respectively. The* red line* (**EB-regularized-LR**, proposed method) is the likelihood-ratio statistic with larger regularization. The six panels from the leftmost to the rightmost columns correspond to the experimental settings of observation lengths $T=1000, 2000, 5000, 10{,}000, 20{,}000,$ and 50,000

**Fig. 3:**
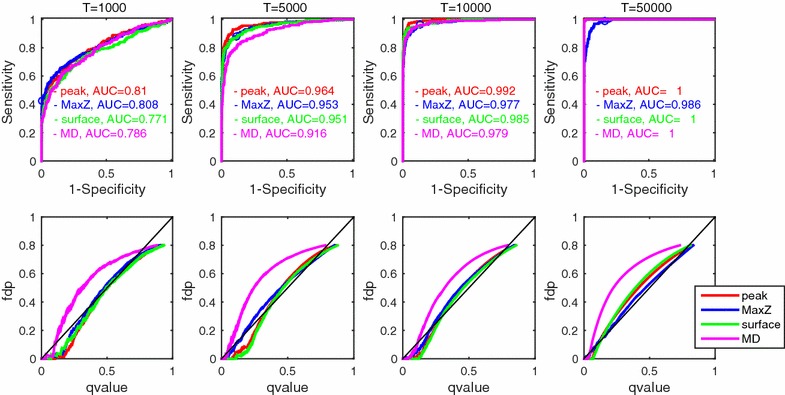
Results of Experiment 1 in which total error and false positive control were compared among four test statistics: *Peak* (*red*), *MaxZ* (*blue*), *Surface* (*green*), and *MD* (*magenta*) (“Shape-related statistics” section). See caption of Fig. 2 for explanation of horizontal and vertical axes of *upper* and *lower panels*

Figures 2 and 3 show the results. The upper panels show the ROC curves with AUC scores in the title, in both of which the higher and the larger are the better ones, respectively. In the lower panels, the false discovery proportion (FDP) is shown for each q value threshold on the horizontal axis; FDP is the proportion of false positives (detected as functionally connected, but where there was in fact no direct connection) in such links that were detected as functionally connected because the corresponding q values were smaller than the threshold on the horizontal axis. The lower panels evaluate the quality of the q value estimation. Because a q value is defined as the FDP estimation, when the FDP is smaller than the q value threshold, the corresponding q value estimation is said to be conservative. From the upper panels of Fig. 2, the ROC curves and the AUC scores of the proposed method (**EB-regularized-LR**, red lines) consistently outperformed those of its un-regularized version (**EB-LR**, blue line), and such a benefit of regularization was prominent especially when the observation length *T* was small. In the lower panels of Fig. 2, the conservativeness of the q value estimation was compared among **EB-LR** (blue solid lines), **CHI2** (blue dotted lines), **DOF** (blue broken lines), and the proposed **EB-regularized-LR** (red solid lines). If a line is close to the diagonal $x=y$ line, the corresponding q value estimation is faithful, and if a line is below the diagonal line, the q value estimation is conservative. We prefer q value estimation that is faithful but conservative; that is, we do not prefer a line that goes over the diagonal line. We found that the empirical Bayesian q values (blue solid lines) were stably good, while a classic chi-square test without DOF adjustment (**CHI2**; blue dotted lines) almost failed to perform false positive control when the observation length *T* was small. When the observation length *T* was large enough, the results depicted by the blue lines were consistent with those reported by [7]. In the lower panels, the FDP values sometimes went over the diagonal line, violating the conservativeness. This violation comes from the estimation variance of q values; a large variance in the q value estimation may arise especially when there is significant correlation between hypotheses, which is the case in the functional connectivity analysis of neuronal networks.

**Fig. 4:**
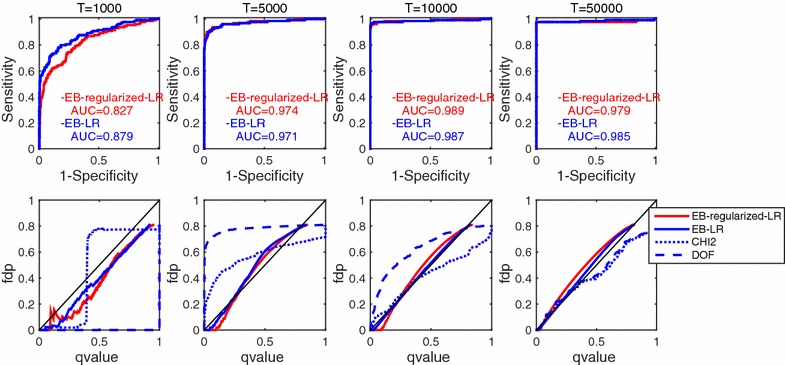
Results of Experiment 2 in which spike trains ware observed from a recurrent network of 15 non-linear conductance-based (Hodgkin–Huxley type) neurons. Notations are identical as in Fig. 2

Figure 3 shows the results of statistical tests employing shape-related statistics (“Shape-related statistics” section). We did not find any substantial differences either in total detection accuracy (upper panels, evaluated in terms of ROC) or false positive control (lower panels, evaluated in terms of faithfulness and conservativeness of the q value estimation) among the statistics of the *Peak* (red lines), the *MaxZ* (blue lines), the *Surface* (green lines), and the *MD* (magenta lines). It is noteworthy that we numerically confirmed the stable false discovery rate control regardless of possibly different null distributions of various test statistics. This result may allow us to select arbitrary test statistics in the proposed empirical Bayesian testing framework. We may expect good ROC by selecting a relevant test statistic that fits particular shapes of true spike response functions although the ROCs were similar between different statistics in the current case partly because we assumed simple spike response function shape such that caused no contradiction between test statistics that we compared here. The ROC curve of *MaxZ* statistic included 95 % CI criteria as a special case (see “Statistical tests for functional connectivity analysis” section), which is specified as a marker ’o’ on the curve (upper panels in Fig 3). We found that the 95 % CI provided a moderate balance between sensitivity and specificity in these cases. However, note that the 95 % CI had no guarantee of conservativeness that EB could provide as q value.

### Experiment 2: a network of non-linear conductance-based neurons

Next, we applied functional connectivity analysis to a model mismatch case. A spike train dataset was obtained by simulating a recurrent network of non-linear conductance-based neurons. Each neuron was modeled by single-compartment Hodgkin–Huxley (HH) equations [25], consisting of sodium, potassium, and leak channels. Thus, the neuronal activities did not follow GLMs. We, however, expect that the proposed false positive control procedure works reasonably well because our framework mainly depends on the empirical null distribution of test statistics built upon a set of surrogate neuron pairs and hence does not depend on the GLM’s fitness.

The simulated recurrent network resembled that in Experiment 1: three layers, each of which had five HH-type neurons. The simulation was conducted using the NEST simulator [26, 27], and the parameters of the HH neurons were set by the default setting of the NEST simulator.

The results are shown in Fig. 4, in which similar behaviors of the statistical tests can be seen to those in Experiment 1. The q value estimation by chi-square tests, **CHI2-LR** (blue broken lines) and **DOF-LR** (blue dotted lines), was substantially poor even when observation length *T* was large. When employing non-linear neurons like the HH-type ones, GLM could no longer well represent their non-linear behaviors, and the model of the alternative hypotheses would contain bias, which was not resolved even after very long observation. Although the GLM-based spike response model had bias, the empirical Bayesian testing with regularized model estimation, **EB-regularized-LR** (red lines), showed reasonably good ROC curves and AUC values (upper panels) and faithful estimation of the q values (lower panels), based on an empirical null distribution constructed by the surrogation method.

**Fig. 5:**
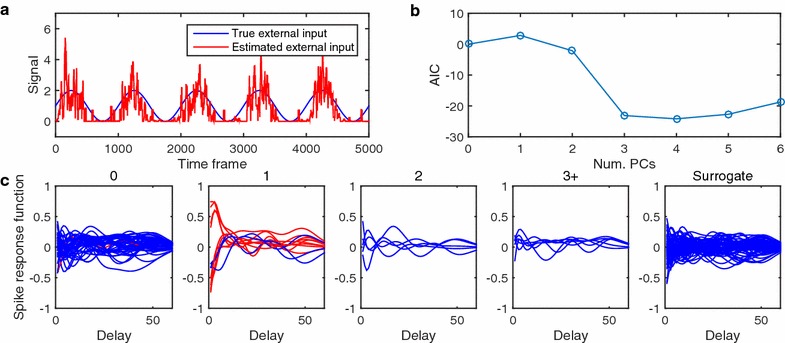
Results of Experiment 3 that considers partial observation with an external input. **a** External input signals. The artificially designed and an estimated signals are shown as *blue* and *red lines*, respectively. 5000 time samples out of 400,000 time samples are shown here. **b** Model selection via AIC criterion. *Vertical* and *horizontal axes* denote AIC criterion and number of external inputs that are involved in the GLM. AIC values are normalized so as to that with no external input is zero. **c** The estimated spike response functions. Five panels correspond to the following categories of neuron pairs; (0) not connected, (1) directly connected, (2) connected via one interneurons, ($3+$) connected via two or more interneurons, and (surrogate) connection from/to surrogate neurons. *Red line* denotes statistically significant directed neuron pairs of $q<0.3$

**Fig. 6:**
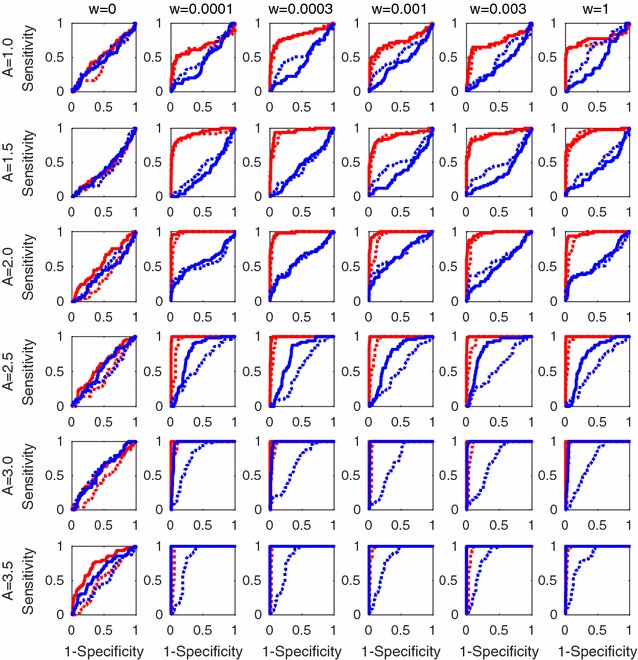
Results of Experiment 4 that assessed functional connectivity detection performances at various spike frequency and connectivity weights with fixing time sample number at $T=2000$. Spike frequencies and connectivity weights are controlled by parameters *A* and *w*, respectively. Rows and columns of the 36 panels denote variations of *A* and *w* values, respectively. In each panel, we compared four ROC curves; *red* and *blue line color* denote estimation methods of GLM, L2 regularization and variational Bayes estimation, respectively. *Real* and *dotted line* styles denote *Peak* and *MaxZ* statistics, respectively. The combination of GLM with L2 regularization and Peak statistic performed the best for most of the 36 panels

**Fig. 7:**
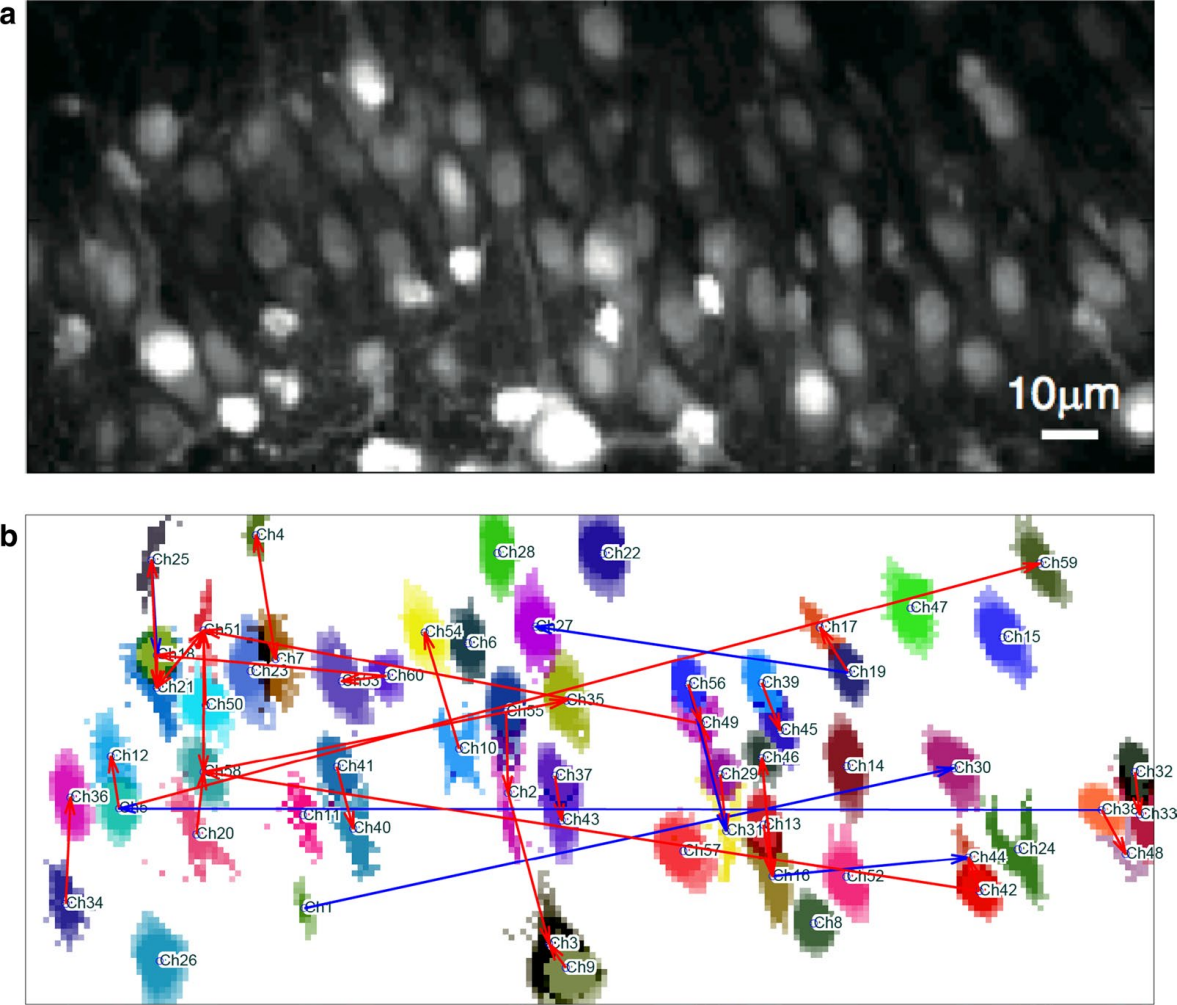
Analysis of calcium imaging data. **a** Averaged calcium fluorescent image of a rat CA3 region. **b** 60 neurons (ROIs) and 43 probable directed connections (with the 43 smallest p values, $\hbox {q} < 0.2$) between the 60 neurons. *Red* and *blue arrows* denote putative facilitatory and suppressive connections, respectively. Statistical properties of the empirical Bayesian testing

**Fig. 8:**
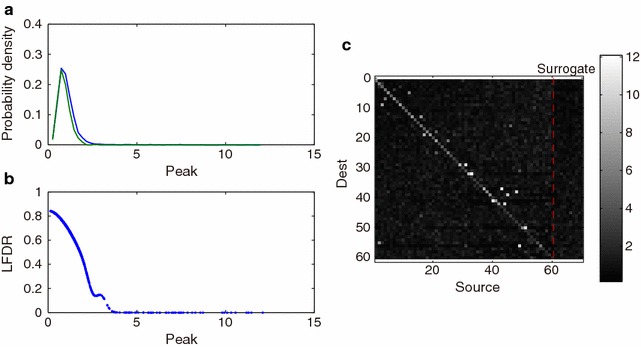
Statistical properties of the empirical Bayesian testing. **a** The estimated distribution (*blue*) and surrogate null distribution (*green*) of the test statistic, *Peak*. The *blue curve* denotes the estimated total distribution, which is a normalized histogram of the estimated statistic of all possible pairs of observed neurons. The *green curve* denotes the estimated null distribution, which is a normalized histogram of the estimated statistics of the pairs of the observed and surrogate neurons. **b** The estimated local false discovery rate. *Vertical* and *horizontal axes* denote local false discovery rate and the test statistic (*Peak*). Each *blue* point corresponds to a pair of source and destination neurons. **c** A matrix of the estimated test statistic, *Peak*. *Rows* and *columns* denote destination and source neurons, where the source neurons include ten surrogate neurons. The *color code* denotes the value of the test statistic, *Peak*

**Fig. 9:**
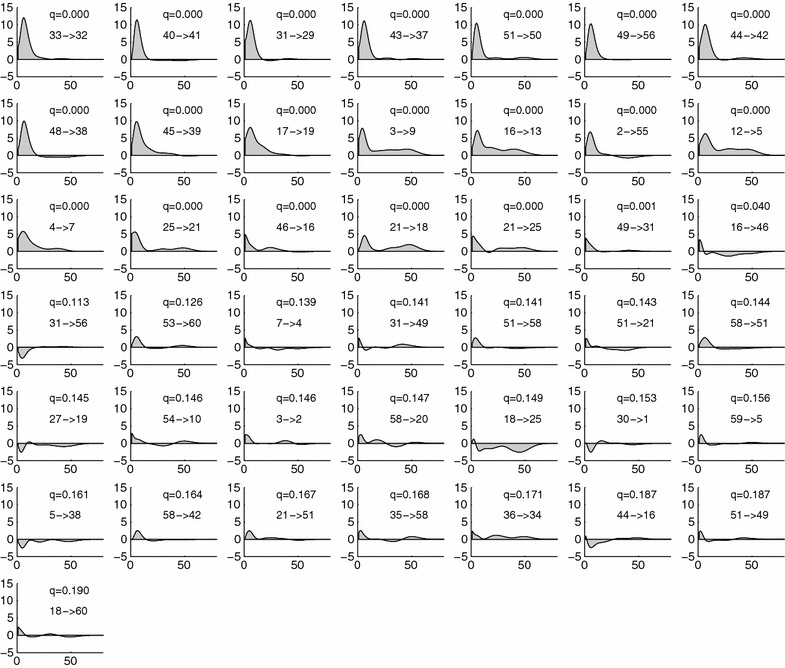
Spike response functions of 43 top significant functional connections, arranged from the* top-left* to* bottom-right panels* in ascending q value order. In each panel, a pair of source and destination neurons’ code numbers and the corresponding q value are shown. *Horizontal* and *vertical axes* denote the lag-time *s* [time sample] and the spike response function, respectively, where 1 sample corresponds to 10 ms

**Fig. 10:**
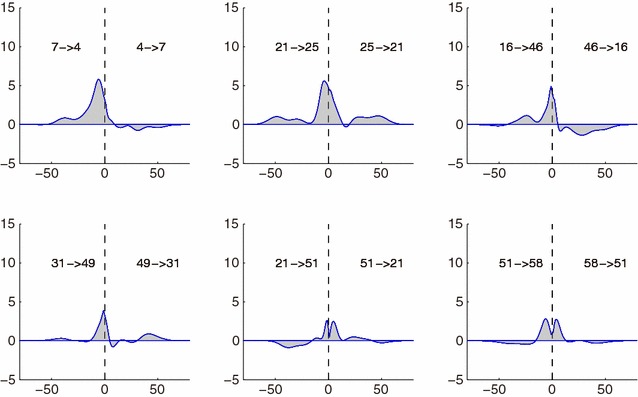
Spike response functions of six pairs of significant bi-directional connections. In each panel, both directed spike response functions are shown; for $0< s < 80$, each spike response function represents $R_{ic}(s)$, while for $-80<s<0$, it represents $R_{ci}(-s)$, instead. From the definition of spike response functions $R_{ic}(0) \approx R_{ci}(0)$, which corresponds to the correlation of neurons *i* and *c*

**Fig. 11:**
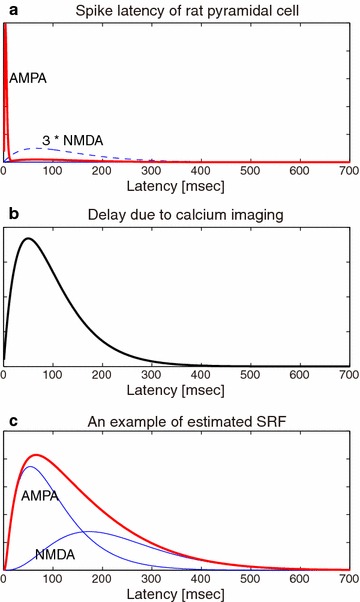
Spike response function and EPSP possibly mediated by AMPA and NMDA receptors. **a** A mixture of fast and slow EPSPs, represented by two gamma distributions peaking at 5 and 50 ms, respectively. According to an electrophysiological study, rat CA1 pyramidal neurons showed sharp, strong and relatively slow EPSPs peaking at around 10 ms and lying over 10–100 ms, respectively [30], which were possibly mediated by AMPA and NMDA receptors. In the mixture, the amount of the slow EPSP was set as small as one third of the blue broken line. **b** The delayed response of the calcium indicator induced by the membrane voltage was represented by a convolution kernel, an alpha function of the delay time of 50 ms. **c** By convolving the convolution kernel **b** to the typical EPSP (membrane voltage), we have a typical response function obtained by our functional connectivity analysis. Contributions in the response function from AMPA- and NMDA-mediated EPSPs are displayed as *blue lines*

### Experiment 3: partial observation case

We applied the proposed method to a partial observation case involving external input. We generated an artificial spike train by a simulation of 400,000 time samples using the same artificial neural network as the ones in Experiments 1 and 2 again. In the simulation, we added an artificial external input signal (Fig 5a) to all neurons, where connection weight from the external input to the neurons were set at random positive values.

In order to consider partial observation, we regarded 7 out of 15 neurons were considered as non-observed neurons, and spike response functions between 8 observed neurons, namely those of 8 times 8 pairs of pre- and post-synaptic neurons, were estimated by our procedure.

The external input was estimated by applying moving average filter of window size 20 and PCA. In Fig 5a, we show the estimated external input denoted as red line. We compared AIC values of seven models with 0,1,..., and 6 external inputs, and found that the model with one external input was the best (Fig. 5b). The estimated spike response functions are shown in Fig 5c, where those function shapes corresponding to the 8 times 8 directed neuron pairs were divided into the following four cases: (0) not connected, (1) directly connected, (2) connected via one interneuron, and (3+) connected via two or more interneurons. In addition, we also show spike response functions corresponding to a pair of a surrogate and any observed neurons, that is called a category (surrogate).

The spike response functions were estimated as significant ($q<0.3$) for a large part of direct connection, red lines in case (1) in Fig 5c. On the other hand, the estimated spike response functions of indirect connections, cases (2) and (3+), were very weak and difficult to be distinguished from those of cases (0) and (surrogate). The results were identical when the number of principal component was set at zero, two, and more (data not shown).

### Experiment 4: spike frequency, connection weight, and detection accuracy

How do different spike frequency and connection weight affect the detection accuracy in case of information shortage? Does the proposed framework keep its advantage against sparse estimation methods in these cases?

We generated an artificial spike train of 2000 time samples by a simulation of a simple network involving three neurons, 1, 2, and 3. The network had two directed facilitative connections from 2 to 1 and from 2 to 3, whose connection weights, or peaks of true spike response functions, were 1.0 and *w*, respectively. Here we compared six values of *w* in $\{0, 0.003, 0.001, 0.03, 0.01, 1\}$. All the three neurons had the same background activity level *A*, that was $R_{i0}$ in equation (2), which controls total spike frequency. We compared six values of *A* in $\{1.0,1.5,2.0,2.5,3.0,3.5\}$. As the network size is small, we repeated the same simulation 50 times with different random seeds and averaged the performances in order to draw stable ROC curves.

We compared two GLM estimation methods with the L2 regularization with an arbitrary setting of $\lambda =0.1$ and with the variational Bayesian (VB) estimation [15]. VB approximates a hierarchical Bayesian model with an automatic relevance determination (ARD) technique that determines sparse solution so as to optimize model fitting. For test statistics, we compared *Peak*, as a representative of the simplest cases, and *MaxZ*, as a case that is equivalent to the application of 95 % confidence interval [15].

Figure 6 shows the results of Experiment 4. The 36 panels correspond to the $6 \times 6$ variations of simulation setting parameters, *w* and *A*. Four ROC curves in each panel illustrate detection powers of the connection from 2 to 3 of weight *w* by the four methods, L2 with *Peak*, L2 with *MaxZ*, VB with *Peak*, and VB with *MaxZ*. In total, we found that L2 prominently outperformed VB in most of the cases. We also found that larger *A* tend to improve the detection accuracy as we expected. Interestingly, change of connection weight *w* did not affect much to the detection accuracy except for the case showing chance level $w=0$. Test statistic *Peak* performed a little bit better than *MaxZ* indicating non-omnipotency of the confidence interval criteria although we could not conclude general superiority of *Peak* statistic, neither.

Note that the prominent performance of L2 against those of VB is consistent to the result of Experiment 1. It, however, does not stand for the general inferiority of VB. Further discussion with general comparison will be found in “Alternative options in smoothing and regularization” section.

### Analysis of calcium imaging data

We applied functional connectivity analysis with GLM-based spike response models to a real calcium imaging dataset. A high-throughput calcium imaging experiment was performed on a cultured slice of the CA3 region of rat hippocampus [11]. Using the Nipkow disc, the sampling frequency was as high as 100 Hz, and the number of time samples was $T=60,000$; that is, the observation time was 10 min. During preprocessing, 170 regions of interest (ROIs), each of which showed its individual activity, and the corresponding time-courses of the fluorescence level were obtained. A visual inspection found that each ROI mostly corresponded to a neuron. Then, for each fluorescence time-course, we detected the peak timings by applying a fixed template that represented a calcium profile associated with a single spike. A single detected peak may not correspond to a single real neuronal action potential; actually, there were some cases in which only one spike was detected for burst-like calcium activity. We simply regarded such burst activity as a single spike because their number was small. More detail on the above preprocessing is shown in the Additional file 1. We selected 60 neurons out of the 170 ROIs that showed frequent spike activities and found that the highest spike frequency was 20 times larger than the lowest one within them. After this preprocessing, we evaluated the functional connectivity between the 60 neurons and their statistical significance using our method empirical Bayesian testing after the regularized parameter estimation of the GLM-based spike response model.

For each neuron pair, the spike response function of at most an 80 samples (800 ms) time-lag was estimated. We prepared ten surrogate neurons and assumed two channels of external inputs, which were estimated by applying PCA analysis (“Estimation of external input” section) to all of the 60 neurons’ activities. We found that the false positive control based on shape-related statistics worked as well as the likelihood test statistic, so the q value threshold was determined using the *Peak* statistic (“Shape-related statistics” section).

Figure 7 shows the result of the functional connectivity analysis, in which 43 probable directed connections are shown by arrows. The number of connections (43) was determined so that the estimated false discovery rate was 0.2. In this figure, some neuron pairs connected by red arrows (putative facilitatory connections) and being located close to one another may be single neurons segmented into two parts (ROIs), such as soma and axon hillocks. Even if such pairs are removed, the detected functional connectivity still tends to connect two neurons near each other. There were five neuron pairs connected by blue arrows (putative inhibitory connections) being located distant to each other.

Statistical properties of the proposed empirical Bayesian testing for this dataset are shown in Fig. 8. The probability distribution of a test statistic is a mixture (a weighted sum) of unknown null and alternative distributions, which would correspond to the left mass and the right tail of the density function shape in panel (a), respectively. The surrogate distribution of the test statistic (green curve) fits well to the left mass part of the empirical distribution (blue curve), indicating that the surrogate distribution well simulated the unknown null distribution. The local false discovery rate (panel b) shows that the estimated total proportion of null connections was $\pi _0=0.83$ and that the local false discovery rate was smaller than 0.2 when the statistic was larger than 2.5. The significant pairs of neurons are shown as white dots in the source/destination matrix (panel c), so that their degrees of significance are seen by comparing to the background noise level in the surrogate area of the matrix.

The spike response functions of significant functional connectivity are shown in Fig. 9 in ascending order of q values. Many response functions of the top significant functional connectivity had a positive peak at lag-times of around 8 samples ($\approx 80$ ms), and some response functions included a tail around the 50th sample ($\approx 500$ ms), which can be consistently considered as EPSPs possibly mediated by AMPA and NMDA receptors (see Discussion for details). Moreover, obvious continuity of response functions between pre-post connections, $R_{ic}(s)$, and their reverse ones, $R_{ci}(s)$, was found for many pairs of neurons, (*i*, *c*), in Fig. 10. This continuity (at $s=0$) occurs partly because it reflects the correlation of neurons *i* and *c*. Such correlation might have been overestimated due to jitter in spike timing detection; in some cases, neuron *i* emits a spike slightly earlier than that of neuron *c*, and in other cases, neuron *c*’s spike can be earlier, because of the jitter in the binning.

## Discussion

### Information shortage and detection performance

To detect functional connectivity, we need sufficient events of delayed spike pairs at the corresponding pair of neurons. Moreover, the number of events should exceed the background level of spontaneous activity of the post-synaptic neuron. Thus, various factors can reduce detection performance via information shortage; shorter observation time length, lower total spike frequency, weaker connection weight, an unknown external input to post-synaptic neuron, high background activity of post-synaptic neuron, and so on. Any of these factors unavoidably exists in real situations and consideration of information shortage is always vital for any case even when the observation time length is fairly long. This point of view motivated the current study. And, a part of them were demonstrated in Experiments 1 and 4 in this paper.

### Alternative options in smoothing and regularization

Recently, two distinct approaches have been proposed for functional connectivity analysis from the neuronal spike train data. In the first approach, hierarchical Bayesian methods were applied to GLM-based models to obtain sparse solutions [4, 13, 15, 28]. A prior in the hierarchical Bayesian setting allows the statistical estimation of GLM-based models to prefer sparsely connected networks, such as reflecting the sparse structure of neuronal networks. The sparseness also reduces the complexity of each spike response function. The second approach was based on Granger causality with a likelihood-ratio test [7]. Functional connectivity analysis based on Granger causality can be performed for any regression model; the authors used the simplest AR model without any smoothing kernel or regularization to enable the null distribution of the likelihood-ratio test to be analytically calculated.

These two approaches are based on different statistical approaches but share the same objective of functional connectivity analysis; combining these approaches has options, such as, how to introduce regularization and how to define null and alternative hypotheses in the likelihood-ratio test. In this study, we evaluated several reasonable options in terms of two criteria in statistical testing: (1) appropriate false positive control, which is based on good estimation of the false discovery rate in multiple simultaneous testing, and (2) high detection accuracy which is determined by increasing the number of true positives while fixing the level of false positives.

Further comparison to Chen et al. (2011) [15] may better illustrate the difference between the two approaches. Their work proposed two distinct ideas, automatic-relevance-determination (ARD) with variational Bayes (VB) to estimate connection weights of GLM and a use of 95 % confidence interval (CI) criteria to determine the connection. Their ARD with VB technique estimates sparse model as like the L1 regularization does with optimizing sparseness hyperparameter, and thus the result tends to be as good as the best among the L1 of various regularization parameters. The use of the 95 % CI may resemble our work in the consideration of false positive risk control. However, note that both of these ideas were designed based on the first approach for the purpose of fitting a generative model to the observed data rather than to detect functional connectivity. The best model, measured by likelihood, is not necessarily the best model, measured by ROC curve. Furthermore, the gap between these two criteria tends to be larger at information shortage. We showed this contradiction in Fig 1 of Experiment 1 and more prominently in Experiment 4. Our approach remarkably outperformed the VB if the performance is measured by ROC. Consequently, we recommend choosing appropriate approach with determining purposes in advance to the analysis.

### Model mismatch bias

Model mismatch bias is an important issue that needs to be addressed here. Any Bayesian methodologies using prior probability to improve estimation performance always, at least implicitly, presume that the parametric model includes the truth. Although such a presumption can actually improve the estimation performance in ideal situations without any model mismatch, most models include a mismatch to the reality to a greater or lesser extent. There is no guarantee that the GLM represents actual neural behaviors well enough even with the best setting of the model parameters. However, our framework is based on statistical testing that allows model mismatch to some extent. Namely, the alternative model does not necessarily include the truth because the functional connectivity is determined by rejection of the corresponding null hypothesis. Better and worse alternative models lead to decrease and increase in false negative rate, but do not affect false positive control. The false positive is well controlled if the surrogate distribution well simulates the null process, that is, spike response between functionally unconnected neurons in our case. Experiment 2 (“Experiment 2: a network of non-linear conductance-based neurons” section) showed a reasonable performance in a typical model mismatch case where the data were generated by the Hodgkin–Huxley equations and analysis was done based on the GLM model. The analysis of calcium imaging data (“Experiment 4: spike frequency, connection weight, and detection accuracy” section) suggested that the surrogate distribution well simulated the null part of the distribution of the *Peak* statistic. We thus conclude that our method can perform conservative statistical testing in many practical cases including that of model mismatch.

### Computational cost

Computational cost for the GLM parameter estimation is proportional to the observation length and to the square of the number of neurons. When we add surrogate neurons for the empirical Bayesian testing, it increases the effective number of possible connections and causes a corresponding increase in computational cost. Note that the number of surrogate neurons $C_{{\mathrm{S}}}$ may not be so large when the real neuron number *C* is large because the variance in the statistical test depends on the number of null samples of their inter-connections, $C_{{\mathrm{S}}}C$. We set $C_{{\mathrm{S}}}$ so that the number of null samples was around $C_{{\mathrm{S}}}C=300$ in our experiments.

### Partial observation

Partial observation is an important practical issue because it is difficult for many physiological techniques to cover all neurons recruited by the target system; functional connectivity analyzes should cope with this issue.

In experiment 3, we demonstrated that spike response functions of indirectly connected pairs of neurons were far weaker than those of directly connected pairs. We conducted similar experiments with various other settings and found similar results (data not shown), where the variants included different observation length, 10,000, 50,000, and 100,000, different sets of observed and non-observed neurons, different moving average windows in PCA analysis, different strengths of external inputs, and different network structures. We consistently observed the following tendencies:

- When a strong external input exists, it is detected by the proposed procedure with PCA and AIC.
- The proposed procedure with PCA with AIC hardly ever detected a total activity of non-observed neurons as a distinct external input. The effect of non-observed neurons was indistinguishable to a background activity level of each observed neuron.
- Spike response was very weak and not significant for indirect connections.
- Spike response of direct connection was stronger than that of indirect connections but weaker than that in fully observed cases.

In analysis of partial observation case with the GLM model, the total effect of non-observed neurons is roughly summed up into the scalar value of background activity level, which causes severe loss of information compared to the fully observed case. This information loss has probably caused the higher estimation variance of spike response and the lower sensitivity to detect direct and indirect functional connectivities.

In total, Experiment 3 demonstrated an additional reason to consider information shortage that might be present in case of long observation length and to need the proposed procedure with empirical Bayesian test with surrogate neurons to control false positives. Further analysis would be needed as future studies to clarify the effect of non-observed neurons to the estimation variance although it is out of scope of the current study.

### Biological suggestions

When we applied the proposed method to the rat CA3 calcium imaging dataset, we extracted 43 directed connections ($q<0.2$). Many of their response functions exhibited sharp peaks at around 8 samples (80 ms), while some (e.g., $3\rightarrow 9$, $16\rightarrow 13$, and $12\rightarrow 5$) were also accompanied by dull and weak tails lying over 10–50 samples (100–500 ms) (Fig 9). The delayed activation corresponding to the weak tails may reflect sequential spike chains across multiple neurons, which prevail in hippocampal networks and often last for more than 100 ms [29]. More physiologically, the sharp peaks and weak tails may reflect EPSP of rat hippocampal pyramidal neurons revealed by an electrophysiological study; reportedly, EPSP of rat CA1 pyramidal neurons showed sharp strong peaks at around 10 ms and relatively weak tails of 10–100 ms delay [30], which were putatively mediated by AMPA and NMDA receptors. In fact, when we convolved an alpha function with a delay time of 50 ms (Fig. 11b), which represents a chelating property of our calcium indicator (Oregon Green 488 BAPTA-1AM), to the mixture of the fast AMPA-mediated EPSP and the late NMDA-mediated EPSP (Fig. 11b), we could reproduce a typical shape of our response functions (Fig. 11c). Changes in the mixture of AMPA- and NMDA-mediated EPSPs would produce various kinds of response functions. Therefore, the variety in the response functions found by our data-driven functional connectivity analysis (Fig. 9) may have reflected to some extent the difference in the receptor distributions between the pyramidal neurons in the rat CA3 circuit.

There were two prominent negative responses, $16 \rightarrow 46$ and $18\rightarrow 25$, that also included positive peaks (Fig 9). The positive peak of $18\rightarrow 25$ was weak and could not be statistically significant, while that of $16 \rightarrow 46$ was still prominent. Seeing the response function of the counter direction $46\rightarrow 16$, the positive peak in $16\rightarrow 46$ was considered to be an artifact coming from the significant positive peak in $46\rightarrow 16$ (Fig 10). There were no other bi-polar responses that were significantly strong enough.

## Conclusions

In this study, we presented a new combination of GLM-based spike response modeling and empirical Bayesian testing to perform functional connectivity analysis between neurons. Even when the observation period was relatively short, our method showed reasonably good detection accuracy while keeping good false positive control. Empirical Bayesian testing effectively estimated the q values for multiple simultaneous hypotheses testing, leading to good false positive control. A regularized but non-sparse estimation for the GLM-based spike response model improved the detection accuracy. Conventional testing procedures have suffered from difficulties in approximating the false discovery rate, particularly when a likelihood-ratio statistic is biased, for example, when with a regularization; our approach based on empirical Bayesian testing is a reasonable solution to the difficulty. Our new method’s contribution is prominent, especially when the sample size is relatively small and there are short observation periods like in many in vivo and *ex vivo* imaging experiments. A typical example can be seen in functional multi-neuron calcium imaging, where high-temporal resolution restricts the observation length. In addition, we found that empirical Bayesian testing on arbitrary statistics that represent the shapes of spike response functions attained a similar performance to that using the well-established likelihood-ratio statistic. This finding is important for increasing computational efficiency, because the likelihood-ratio statistics must be calculated after fitting GLMs for all pairs of null and alternative hypotheses, and hence are computationally heavy.

When applied to a functional multi-neuron calcium imaging dataset from a rat hippocampal CA3 region, we found significant functional connections that are possibly mediated by AMPA and NMDA receptors.

Accordingly, our method exhibited reasonably good functional connectivity results even from relatively short observation times and could become a powerful statistical tool in studies of connectomics.

## Additional file

10.1186/s12868-016-0255-x Supplementary document.

## Abbreviations

GLM: generalized linear model
SRF: spike response function
AR: auto-regression
EPSC (IPSC): excitatory (inhibitory) postsynaptic current
PCA: principal component analysis
AIC: Akaike’s information criterion
CI: confidence interval
ROC: receiver operating characteristic curve
AUC: area under the ROC curve
FDP: false discovery proportion
FDR: false discovery rate
HH: Hodgkin–Huxley
VB: variational Bayesian
ARD: automatic relevance determination
ROI: region of interest

## References

[1] Sporns O (2012). Discovering the human connectome.

[2] Stevenson IH, Körding KP (2011). How advances in neural recording affect data analysis. Nat Neurosci.

[3] Valdes-Sosa PA, Roebroeck A, Daunizeau J, Friston K (2011). Effective connectivity: influence, causality and biophysical modeling. NeuroImage.

[4] Stevenson IH, Rebesco JM, Miller LE, Körding KP (2008). Inferring functional connections between neurons. Curr Opin Neurobiol..

[5] Pillow JW, Shlens J, Paninski L, Sher A, Litke AM (2008). Spatio-temporal correlations and visual signaling in a complete neuronal population. Nature.

[6] Stephan KE, Harrison LM, Kiebel SJ, David O, Penny WD, Friston KJ (2007). Dynamic causal models of neural system dynamics: current state and future extensions. J Biosci.

[7] Kim S, Putrino D, Ghosh S, Brown EN (2011). A Granger causality measure for point process models of ensemble neural spiking activity. PLoS Comput Biol.

[8] Stetter O, Battaglia D, Soriano J, Geisel T (2012). Model-free reconstruction of excitatory neuronal connectivity from calcium imaging signals. PLoS Comput Biol.

[9] Isaac JTR, Hjelmstad GO, Nicoll RA, Malenka RC (1996). Long-term potentiation at single fiber inputs to hippocampal CA1 pyramidal cells. Proc Natl Acad Sci USA.

[10] Liao D, Hessler NA, Malinow R (1995). Activation of postsynaptically silent synapses during pairing-induced LTP in CA1 region of hippocampal slice. Nature.

[11] Takahashi N, Oba S, Yukinawa N, Ujita S, Mizunuma M, et al. High-speed multineuron calcium imaging using Nipkow-type confocal microscopy. Curr Protoc Neurosci. 2011;2:2(14). 10.1002/0471142301.ns0214s57 21971847

[12] Stevenson IH, Rebesco JM, Hatsopoulos NG, Haga Z, Miller LE, Körding KP (2009). Bayesian inference of functional connectivity and network structure from spikes. IEEE Trans Neural Syst Rehabil Eng.

[13] Gerwinn S, Macke JH, Bethge M (2010). Bayesian inference for generalized linear models for spiking neurons. Front Comput Neurosci..

[14] Pillow JW, Ahmadian Y, Paninski L (2011). Model-based decoding, information estimation, and change-point detection techniques for multi neuron spike trains. Neural Comput.

[15] Chen Z, Putrino D, Ghosh S, Barbieri R, Brown EN (2011). Statistical inference for assessing neuronal interactions and functional connectivity with sparse spiking data. IEEE Trans Neural Syst Rehabil Eng.

[16] Song D, Wang H, Tu CY, Marmarelis VZ, Hampson RE, Deadwyler Sa, Berger TW (2013). Identification of sparse neural functional connectivity using penalized likelihood estimation and basis functions. J Comput Neurosci.

[17] Quinn CJ, Coleman TP, Kiyavash N, Hatsopoulos NG (2011). Estimating the directed information to infer causal relationships in ensemble neural spike train recordings. J Comput Neurosci.

[18] Eldawlatly S, Jin R, Oweiss KG (2009). Identifying functional connectivity in large-scale neural ensemble recordings: a multiscale data mining approach. Neural Comput.

[19] Nakae K, Ikegaya Y (2014). A statistical method of identifying interactions in neuron-glia systems based on functional multicell Ca2+ imaging. PLoS Comput Biol.

[20] Principe JC, de Vries B, de Olivieira PG (1993). The gamma filter—a new class of adaptive IIR filters with restricted feedback. IEEE Trans Signal Process.

[21] Tomioka R, Suzuki T, Sugiyama M. Super-linear convergence of dual augmented Lagrangian algorithm for sparse learning. J Mach Learn Rese. 2011;12:1537–86. https://github.com/ryotat/dal.

[22] Bishop CM (2006). Pattern recognition and machine learning.

[23] Fisher RA (1922). On the mathematical foundations of theoretical statistics. Philos Trans R Soc Lond Ser A..

[24] Storey JD (2002). A direct approach to false discovery rates. J R Stat Soc Ser B.

[25] Hodgkin AL, Huxley AF (1952). A quantitative description of ion currents and its applications to conduction and excitation in nerve membranes. J Physiol.

[26] Diesmann M, Gewaltig MO. NEST: an environment for neural systems simulations. In: Plesser T, Macho V, editors. Forschung und wisschenschaftliches Rechnen, Beitrage zum Heinz-Billing-Preis 2001, Vol. 58 of GWDG-Bericht, Gottingen, Ges. für Wiss. Datenverarbeitung; 2002, p. 43–70.

[27] Davison AP, Brüderle D, Eppler J, Kremkow J, Muller E (2008). PyNN: a common interface for neuronal network simulators. Front Neuroinformatics.

[28] Mishchenko Y, Vogelstein T, Paninski L (2011). A Bayesian approach for inferring neuronal connectivity from calcium fluorescent imaging data. Ann Appl Stat.

[29] Sasaki T, Matsuki N, Ikegaya Y (2014). Interneuron firing precedes sequential activation of neuronal ensembles in hippocampal slices. Eur J Neurosci.

[30] Fricker D, Miles R (2000). EPSP amplification and precision of spike timing in hippocampal neurons. Neuron.

