# Supplementary material for: Empirical Bayesian significance measure of neuronal spike response
Source: BMC Neurosci. 2016 May 21;17:27. doi: 10.1186/s12868-016-0255-x (PMC4875706; doi:10.1186/s12868-016-0255-x)
Supplement: Supplementary file 1 — 10.1186/s12868-016-0255-x Supplementary document. [file 12868_2016_255_MOESM1_ESM.pdf]

Appendix to Empirical Bayesian significance measure of neuronal spike response

Shigeyuki Oba, Ken Nakae, Yuji Ikegaya, Shunsuke Aki, Junichiro Yoshimoto, and Shin Ishii

April 30, 2016

1 Preprocessing

In the analysis of calcium imaging data, we obtained spike activities of target neurons from a raw time-lapse movie of calcium imaging through the following preprocessing [1].

1. ROI segmentation. Calculate variance at each pixel along the time course, apply a spatial smoothing filter, detect peaks, determine circular ROIs of radius of 3 pixels around the detected peaks, and refine the ROI shapes by spatio-temporal factor analysis.

2. Raw time course calculation. Calculate spatial average of fluorescence of pixels in each ROI.

3. Baseline subtraction. Calculate baseline by smoothing the raw time course in which outliers are omitted. Subtract the baseline to obtain the signal time course. Here, an outlier was detected when the distance from the corresponding baseline was larger than a standard deviation of the inlier values. This outlier detection was recursively applied with updating the baseline and standard deviation until convergence.

4. Spike detection. Detect peaks in the signal time course obtained above, and each peak was regarded as a spike when its intensity was larger than a certain threshold.

2 A detailed procedure of empirical Bayesian testing

In multiple simultaneous testing, like in functional connectivity analysis, false positive control based on q-value estimation is important. According to empirical Bayesian testing, the q-values of all the hypotheses that are tested simultaneously are calculated based on the set of empirical null samples of the test

statistic rather than on the theoretically obtained null distribution. The set of empirical null samples is generated by a specially designed null generative model. See Section 3.2 of the main text for more details about generating null samples in our particular application.

In this appendix section, we explain the detailed procedure to calculate q-value q_i of the ith hypothesis based on the set of test statistics $\{x^{(i)}|i = 1, ..., D\}$ for all the hypotheses in multiple simultaneous testing and the generated B null samples of test statistics $x_0^{(1)}, ..., x_0^{(B)}$.

We assume that the test statistic x arises from either the null or alternative distribution, that is, from their combination:

$$p(x) = \pi_0 p_0(x) + (1 - \pi_0)p_1(x), \tag{1}$$

where $p_0(x)$ and $p_1(x)$ are the null and alternative distributions, respectively, and π_0 is a prior probability with which x arises from the null distribution.

We estimated density ratio $r(x)$

$$r(x) = \frac{Bp_0(x)}{Bp_0(x) + Dp_1(x)} \tag{2}$$

from the empirical samples using kernel logistic regression (KLR) that will be explained below. The estimated density ratio is denoted as $\hat{r}(x)$.

We performed a conservative estimation of the local false discovery rate:

$$\hat{\text{lfdr}}(x) = \frac{D\hat{\pi_0}}{B} \frac{\hat{r}(x)}{1 - \hat{r}(x)}. \tag{3}$$

$\hat{\pi_0}$ is a conservative estimation of π_0 that was calculated as

$$\hat{\pi_0} = \frac{B}{D} \frac{1 - \bar{r}}{\bar{r}}, \tag{4}$$

where $\bar{r}$ is an average of the κB largest values of $\hat{r}(x_0^{(b)})$ among $b = 1, ..., B$. Here, constant $0 < \kappa < 1$ was set arbitrarily so that $p_1(x) << 1$ for $x > x_0^{(b')}$, where $x_0^{(b')}$ is the κB-th largest value among $x_0^{(b)}$, $b = 1, ..., B$.

Finally, the q-value of the ith hypothesis was calculated by

$$q_i = \frac{1}{D^{(i)}} \sum_{i' \in I_i} \hat{\text{lfdr}}(x^{(i')}), \tag{5}$$

where the summation was taken for sample indices $i' \in I_i$. Here, I_i is the index set of such samples that $\hat{\text{lfdr}}(x^{(i')}) \leq \hat{\text{lfdr}}(x^{(i)})$ holds and $D^{(i)}$ is the number of sample indices belonging to the set I_i. Eq. (5) means that the q-value is an upper bound of the expected false discovery rate if all the hypotheses i' such that $q_{i'} \leq q_i$ are rejected.

We employed KLR to estimate the density ratio $r(x)$ to maximize the likelihood function:

$$L(\beta) = \sum_{b=1}^{B} r(x_0^{(b)}) + \sum_{i=1}^{D} (1 - r(x^{(i)})) + \lambda ||\beta||^2, \tag{6}$$

where

$$r(x) = f\left(\beta_0 + \sum_k \beta_k \phi_k(x)\right), \tag{7}$$

$\phi_k(x)$ $(k = 1, ..., K)$ are arbitrarily prepared kernel functions and $\beta = (\beta_0, \beta_1, ..., \beta_K)$ are their weight parameters. $\lambda > 0$ is a regularization constant. In this study, we used the particular polynomial basis functions $\phi_1(x) = x, \phi_2(x) = x^2, ...$ with an arbitrary number of kernel functions K.

We determined an appropriate value of the regularization coefficient λ and the number of polynomial bases K to maximize the average likelihood (6) by 5-fold cross-validation. Either $K = 3$ or $K = 4$ was used in our experiments.

References

[1] Nakae, K., Ikegaya, Y., *et al.* (2014) A statistical method of identifying interactions in neuron–glia systems based on functional multicell Ca2+ imaging. *PLoS Computational Biology*, 10(11): e1003949.
